# Functionalization of amyloid fibrils via the Bri2 BRICHOS domain

**DOI:** 10.1038/s41598-020-78732-1

**Published:** 2020-12-10

**Authors:** Henrik Biverstål, Rakesh Kumar, Anna Katharina Schellhaus, Médoune Sarr, Nico P. Dantuma, Axel Abelein, Jan Johansson

**Affiliations:** 1 Department of Biosciences and Nutrition, Karolinska Institutet, NEO/Floor 8, Blickgången 16, 141 52 Huddinge, Sweden; 2 Department of Physical Organic Chemistry, Latvian Institute of Organic Synthesis, Riga, 1006 Latvia; 3 Department of Cell and Molecular Biology, Karolinska Institutet, 171 77 Stockholm, Sweden

**Keywords:** Biochemistry, Protein folding

## Abstract

Amyloid fibrils are mechanically robust and partly resistant to proteolytic degradation, making them potential candidates for scaffold materials in cell culture, tissue engineering, drug delivery and other applications. Such applications of amyloids would benefit from the possibility to functionalize the fibrils, for example by adding growth factors or cell attachment sites. The BRICHOS domain is found in a family of human proteins that harbor particularly amyloid-prone regions and can reduce aggregation as well as toxicity of several different amyloidogenic peptides. Recombinant human (rh) BRICHOS domains have been shown to bind to the surface of amyloid-β (Aβ) fibrils by immune electron microscopy. Here we produce fusion proteins between mCherry and rh Bri2 BRICHOS and show that they can bind to different amyloid fibrils with retained fluorescence of mCherry in vitro as well as in cultured cells. This suggests a “generic” ability of the BRICHOS domain to bind fibrillar surfaces that can be used to synthesize amyloid decorated with different protein functionalities.

## Introduction

Proteins can misfold and end up in different types of aggregates, a phenomenon that is linked to many human diseases, but protein assemblies are also widely used in nature as the building blocks of functional materials with many attractive properties^1–3^. Amyloid formation is one particular type of protein folding associated with several severe human diseases^4^ but also with functional assemblies like bacterial surface fibers and spider silk^5, 6^. Amyloid is formed by polypeptide chains in cross β-sheet conformation, i.e. the direction of the β-strands is perpendicular to the fibril axis^7^. The fibrils are thus highly regular but the atomic details of the architectures of determined high-resolution amyloid structures differ substantially^8, 9^. Each amyloid is associated with fibrils made up of one specific protein and although properties such as size, native structure and location differ widely between amyloidogenic proteins, the ultrastructural appearance of amyloid fibrils is similar independent of constituent protein^10^.

Amyloids can be utilized as proteinaceous artificial materials in form of protein nanofibers, which are a new class of nanomaterials with potential applications ranging from global health to materials science, including artificial tissues, scaffolds for cell cultures, biosensors as well as functional biocoatings^2, 11^. The β-sheet structural units that are the backbone of the fibrous structure provide the unique mechanical properties for nanofibers^11, 12^. They can be built from a broad range of different proteins, including proteins from food production^2, 11^. The β-structure of these nanofibers is energetically highly favored, which makes them particularly resistant and difficult to break down for the body’s own clearance systems. These properties open up for functionalizing protein nanofibers in medical applications. A great advantage is that the nanofibers can be equipped with further features by attaching functional sites, such as growth factors, specific biomacromolecules, fluorophores, etc., for novel applications e.g. as cell culture scaffolds or biosensors^11^.

As amyloid fibrils are associated with human diseases, it is conceivable that endogenous defense mechanisms against fibril formation exist, and that a disease state develops when these defense mechanisms are malfunctioning. We have found an endogenous defense—the BRICHOS domain—against amyloid fibril formation and its toxic consequences, which holds potential to be translated to an efficient treatment of aggregation and neurotoxic effects of the amyloid β-peptide (Aβ) in Alzheimer´s disease^13^. The BRICHOS domain differs from other approaches previously tested to treat amyloid disease and was initially found to be expressed together with surfactant protein C (SP-C), one of the most amyloid-prone proteins known^14, 15^. The fact that nature can produce α-helical SP-C led to the discovery that the BRICHOS domain in proSP-C acts as a molecular chaperone that prevents β-sheet aggregation during biosynthesis^16^. Mutations in the proSP-C BRICHOS domain result in amyloid formation of the SP-C part and lethal amyloid lung disease in early childhood^16^. All other known amyloid diseases occur late in life and these observations thus strongly support the hypothesis that the BRICHOS domain is capable of efficiently preventing amyloid formation under normal circumstances. The BRICHOS domain can prevent amyloid formation not only of its physiological clients but also of other amyloidogenic proteins, including Aβ and islet amyloid polypeptide (IAPP) associated with type 2 diabetes^17–19^. Importantly for potential synthetic applications, BRICHOS binds to the surface of Aβ amyloid fibrils, which results in that generation of neurotoxic Aβ species is markedly reduced^20–23^. Here, we investigate whether the ability of BRICHOS to bind to fibrillar surfaces can be harnessed for linking proteins fused to BRICHOS to amyloid fibrils, which could be a versatile way to generate functionalized nanomaterials. We tested this hypothesis by fusing BRICHOS to the red-fluorescent mCherry protein^24^ and confirm the co-localization on fibril surfaces by Thioflavin T (ThT) staining. Following this approach, we demonstrate here that mCherry-linked Bri2 BRICHOS can bind to the fibril surface of six different protein nanofibers, which include four typical human disease-related amyloid fibrils as well as a de novo*-*designed β-structure protein. These results hence provide the basis for decorating protein nanofibers with various functionalities, which can be implemented in designed protein-based materials.

## Results

**Figure 1:**
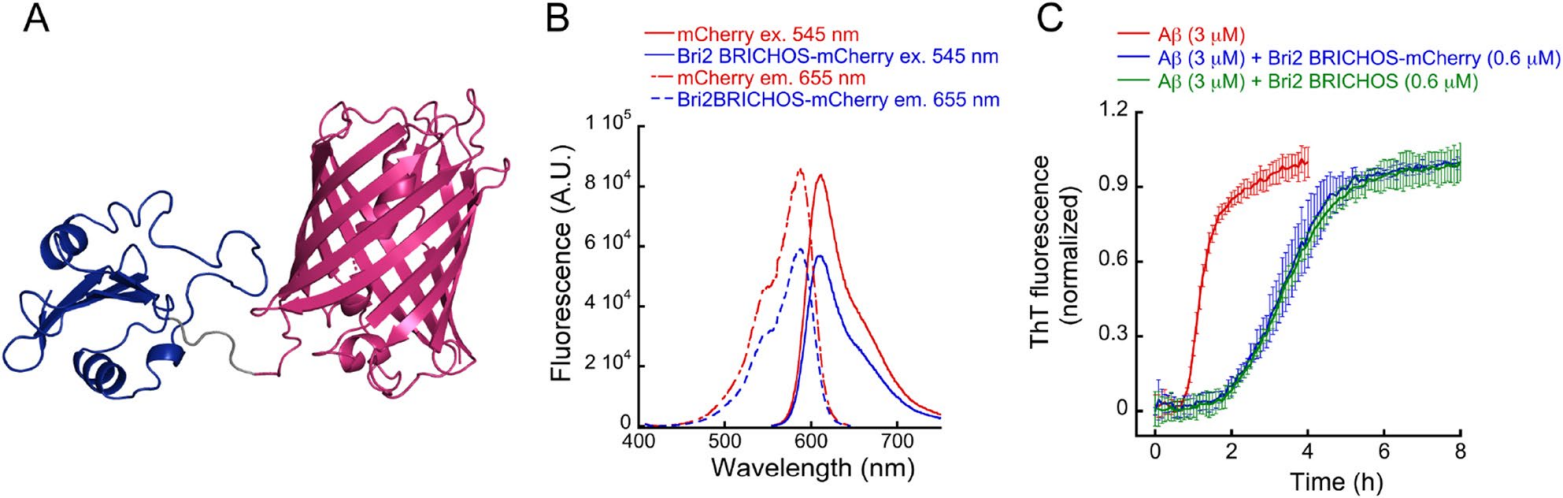
(**A**) Depiction of schematic Bri2 BRICHOS-mCherry structure. Bri2BRICHOS structure model^19^ is represented in blue, mCherry (pdb code 2H5Q) in dark pink and the linker between the two proteins is shown in grey color. (**B**) The fluorescence excitation and emission spectra of Bri2 BRICHOS-mCherry and mCherry alone. The excitation spectra at λ_ex_ = 545 nm and emission spectra at λ_em_ = 655 nm are shown. (**C**) Aggregation traces monitored by ThT fluorescence of 3 µM Aβ_42_ alone (red), 3 µM Aβ_42_ in the presence of 0.6 µM rh Bri2 BRICHOS-mCherry (blue) or 0.6 µM rh Bri2 BRICHOS (green). Error bars represent standard deviations from four measurements.

We cloned the mCherry protein^24^ downstream of human Bri2 BRICHOS, encompassing residues 113–231 in human Bri2^21^ (Fig. 1A) and produced the fusion protein together with a His6 tag and the solubility tag NT* from flagelliform spider silk protein (FlSp)^25^ linked upstream of Bri2 BRICHOS via a thrombin cleavage site. The His6-NT*-Bri2 BRICHOS-mCherry protein was produced in *E. coli*, purified by immobilized metal affinity chromatography (IMAC), and cleaved with thrombin to release recombinant human (rh) Bri2 BRICHOS-mCherry, which was isolated by a second IMAC step. The yield was approximately 100 mg per liter bacterial culture. The fluorescence properties of rh Bri2 BRICHOS-mCherry are very similar to those of mCherry alone (Fig. 1B), which shows that mCherry is functional in the fusion protein. Likewise, the abilities of rh Bri2 BRICHOS to inhibit amyloid fibril formation of Aβ_42_ is retained in the rh Bri2 BRICHOS-mCherry fusion protein as shown by kinetics measured by thioflavin T (ThT) fluorescence (Fig. 1C), indicating that the BRICHOS function is not perturbed by linking it to mCherry. The dual functionalities of rh Bri2 BRICHOS-mCherry motivated us to test whether the fibril binding properties of rh Bri2 BRICHOS^18, 21^ can be used to decorate amyloid fibrils with fluorescent mCherry.

**Figure 2:**
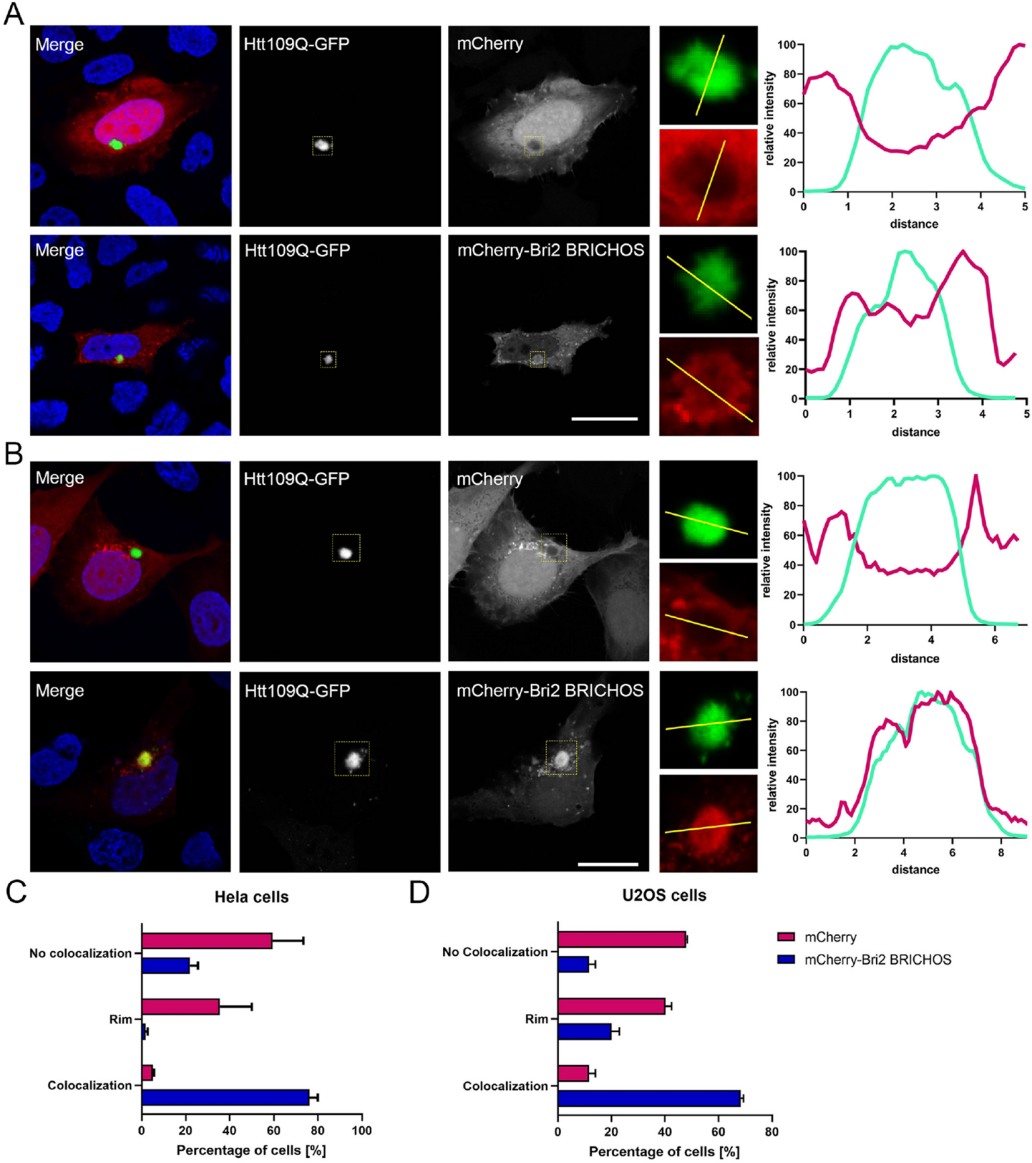
mCherry-Bri2 BRICHOS co-localizes with Htt aggregates. (**A**,**B**) Htt109Q-GFP was transiently co-transfected with either mCherry-Bri2 BRICHOS or mCherry into Hela (**A**) or U2OS (**B**) cells. Cells were fixed 48 h after transfection and chromatin was stained with Hoechst. Line scans through Htt-aggregates show mCherry-Bri2 BRICHOS and mCherry (red) localization at the aggregates (green). Scale bar 20 µm. Manual quantification of localization of mCherry-Bri2 BRICHOS and mCherry at Htt aggregates in Hela (**C**) and U2OS (**D**) cells. Data are presented as mean + SEM of three independent experiments.

The N-terminal fragment of mutant huntingtin (N-mutHtt), which is responsible for the neurodegenerative disorder Huntington’s disease^26^, contains a polyglutamine repeat expansion that renders the protein aggregation prone^27^. When expressed in human cells, N-mutHtt forms amyloid-like fibrils that precipitate in large intracellular inclusions, which can be readily detected by microscopy^28^. Because of this feature, we selected N-mutHtt as a model to explore the ability of Bri2 BRICHOS to interact with amyloidogenic proteins in the cytoplasm, which is a non-physiological intracellular localization for this domain that is part of the secretory protein Bri2(Ref.^29^). Green fluorescent protein (GFP)-tagged N-mutHtt with a 109 amino acid-long repeat (N-mutHtt109Q-GFP) was co-expressed with mCherry or mCherry-Bri2 BRICHOS in human osteosarcoma U2OS cells and human cervix carcinoma HeLa cells and the localization of the proteins were examined by fluorescence microscopy. While mCherry was typically excluded from the GFP-N-mutHtt inclusions or, alternatively localized at the rim of these structures, mCherry-Bri2 BRICHOS was enriched in the inclusions in the U2OS and HeLa cells (Fig. 2). We conclude that the ability of Bri2 BRICHOS to interact with amyloid-like fibrils is an intrinsic feature that is independent of its natural environment.

**Figure 3:**
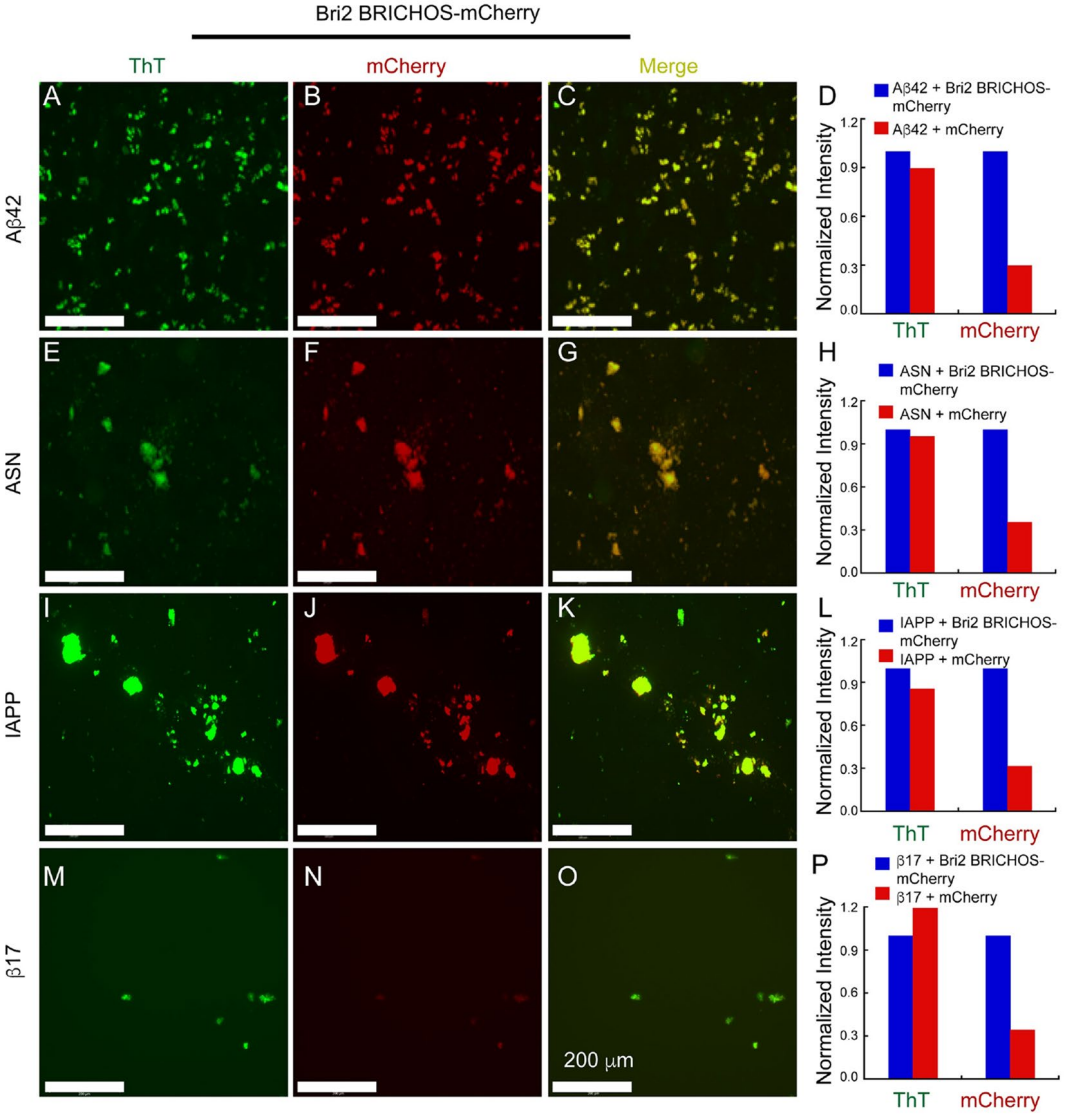
Rh Bri2 BRICHOS-mCherry binding to amyloid fibrils. (**A**,**E**,**I**,**M**) ThT staining of fibrillated Aβ_42_ (**A**), α-synuclein (ASN) (**E**), IAPP (**I**) and β17 (**M**). (**B**,**F**,**J**,**N**) mCherry fluorescence of rh Bri2 BRICHOS-mCherry incubated with fibrillated Aβ_42_ (**B**), ASN (**F**), IAPP (**J**) and β17 (**N**). (**C**,**G**,**K**,**O**), merged images of ThT and mCherry signals. (**D**,**H**,**L**,**P**) normalized signal intensities of ThT (left two histograms) and mCherry (right two histograms) for fibrils decorated with rh Bri2 BRICHOS-mCherry (blue histograms) or mCherry (red histograms). Images of Aβ_42_, ASN, IAPP and β17 fibrils incubated with mCherry are presented in Supplementary Figure 1.

For in vitro analysis of binding properties of rh Bri2 BRICHOS, we generated amyloid fibrils of Aβ_42_, IAPP and α-synuclein, which are implied in Alzheimer’s disease, type II diabetes and Parkinson’s disease, respectively, as well as the de novo-designed β17 protein, according to published protocols^18, 25, 30, 31^. The fibrils were isolated by centrifugation and subsequently incubated with rh Bri2 BRICHOS-mCherry at a concentration that corresponds to 20% of the monomeric amyloid polypeptide concentration, at 37 °C for one hour. After washing, the rh Bri2 BRICHOS-mCherry incubated fibrils were analyzed by fluorescence microscopy. Green fluorescence (corresponding to ThT fluorescence) was observed for all tested amyloid fibrils incubated with rh Bri2 BRICHOS-mCherry. The results confirm amyloid-like properties for the different types of fibrils used. Red fluorescence was observed for rh Bri2 BRICHOS-mCherry suggesting that fusion protein bound to fibrils (Fig. 3). As controls, we incubated the fibrils with mCherry alone. The results show that Aβ42, IAPP, α-synuclein and β17 fibrils all bound rh Bri2 BRICHOS-mCherry, which resulted in fluorescent fibrils, while incubation with mCherry alone only gave marginal staining of the fibrils (Fig. 3 and Supplementary Figure 1). To further confirm that Bri2 BRICHOS-mCherry binds to amyloid fibrils, we tested Aβ_42_E22G (the so-called Arctic mutation related to familial Alzheimer's disease^32^) and a part of the central core region R3 (V306-K317) of Tau (referred to as PHF6) implicated in Tauopathies (e.g. Alzheimer’s disease and Parkinson’s disease), which resulted in similar results (Supplementary Figure 2).

**Figure 4:**
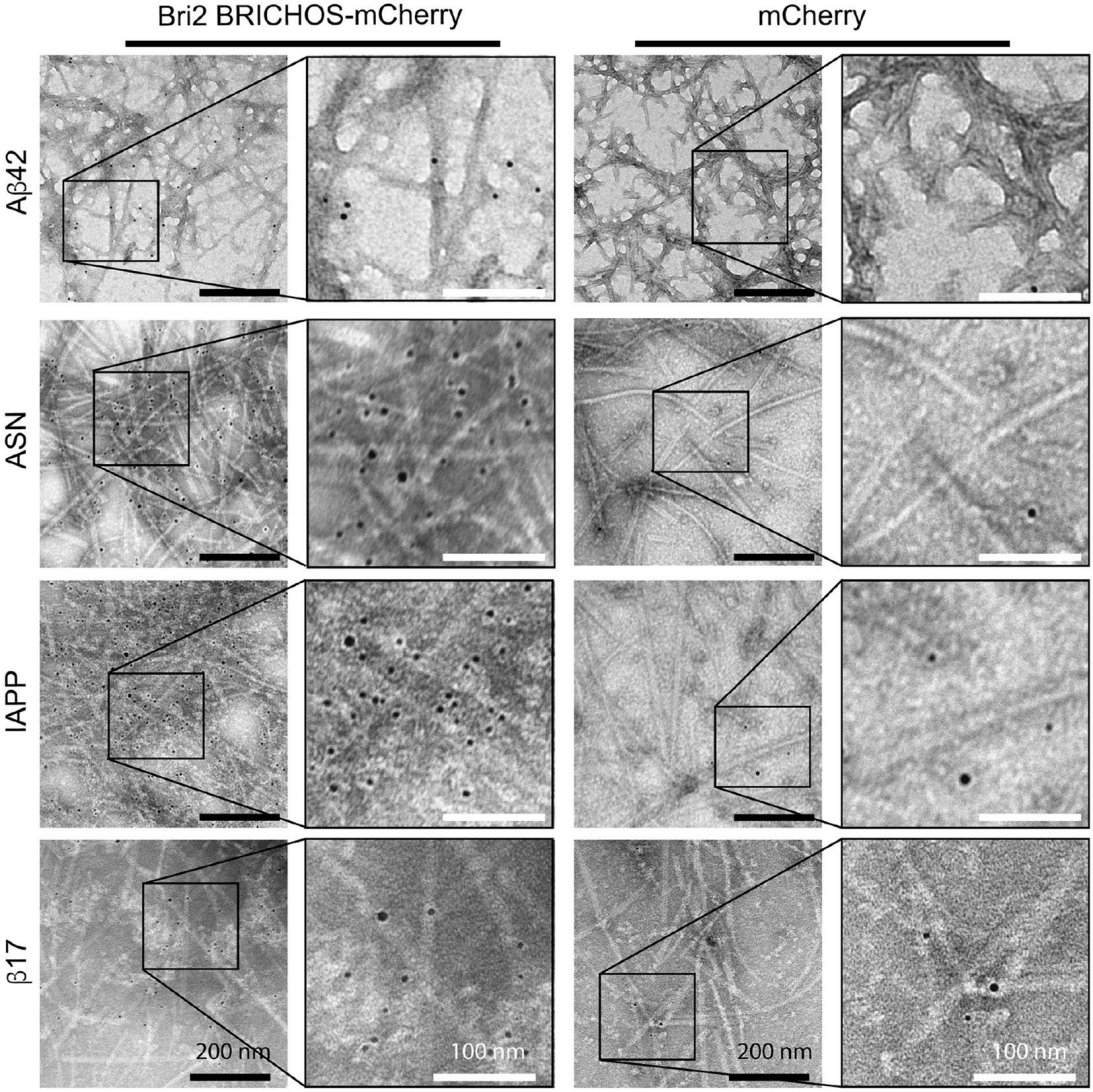
Immunostaining of amyloid fibrils incubated with rh Bri2 BRICHOS-mCherry or mCherry. Gold nanoparticles (5 nm) bound to secondary antibody that binds to primary anti-RFP (red fluorescent protein) antibody are seen on the surface of amyloid fibrils. Specific areas are magnified to show the gold particles.

Next, we examined the localization of rh Bri2 BRICHOS-mCherry on fibrils by immune electron microscopy (EM). To this end, we incubated the rh Bri2 BRICHOS-mCherry decorated fibrils with a primary antibody against mCherry and a secondary antibody bound to 5 nm gold particles and studied them with negative stain EM. The immuno EM data (Fig. 4) confirmed the results from fluorescence studies of the Aβ_42_, IAPP, α-synuclein and β17 fibrils. The gold particles localized on the fibrils for each of the amyloidogenic proteins. While the gold particles gave a strong labeling of the fibrils incubated with rh Bri2 BRICHOS-mCherry, labeling was only sporadically detected with fibrils incubated with mCherry alone.

To quantify binding of rh Bri2 BRICHOS-mCherry and mCherry, we immobilized Aβ_42_ fibrils on a carboxymethylated dextran surface and investigated binding by surface plasmon resonance (SPR). The SPR sensorgrams (Supplementary Figure 3) were used for determining dissociation constants, K_D_ as described previously^33^. Rh Bri2 BRICHOS-mCherry showed strong binding to the immobilized Aβ_42_ fibrils (K_D_ = 75 nM), while mCherry bound significanly weaker (K_D_ = 7 μM). The dissociation constant for rh Br2 BRICHOS-mCherry is in the same range as previously determined for rh proSP-C BRICHOS binding to Aβ_42_ fibrils^22^.

## Discussion

Amyloid-like fibrils are versatile materials that are used in nature for various purposes, and designed nanomaterials built up from regular β-sheets have many potential applications in materials science, biology and medicine. Here we show a potentially “generic” way to decorate amyloid-like fibrils with functional proteins. Our approach is based on the observations from immuno-EM that recombinant BRICHOS binds to the surface of Aβ and IAPP amyloid fibrils^18, 21, 22^. We show that a recombinant fusion protein between human Bri2 BRICHOS and mCherry can be efficiently produced using the solubility tag NT*_FlSp_^25^ derived from the N-terminal domain of spider silk proteins^34^. Rh Bri2 BRICHOS-mCherry shows the same fluorescence properties as mCherry alone, binds to the surface of Aβ_42_ (as well as to fibrils of the Aβ_42_E22G mutant), IAPP, α-synuclein, PHF6 and β17 amyloid fibrils and makes them fluorescent. The binding of rh Bri2 BRICHOS-mCherry to amyloid fibrils in vitro is accomplished simply by co-incubating the fusion protein and the fibrils in aqueous buffer at 37 °C for one hour under quiescent conditions, and specific binding of mCherry tagged Bri2 BRICHOS to amyloid-like inclusions of mutant huntingtin is observed in the cytoplasm of two different cell lines. These conditions will likely be tolerable by other Bri2 BRICHOS fusion proteins and other amyloid-like fibrils and nanomaterials. The approach presented here thus holds potential to be useful for decorating various fibrillar materials. Artificial spider silk-like fibers, with amyloid-like properties, are thought to have attractive properties for generation of novel biomaterials^35, 36^ and can form fibers also when linked genetically to other proteins^37^. One drawback with fusing proteins to a fiber-forming protein is that the added protein needs to withstand conditions required for silk fiber formation, which can entail for example presence of organic co-solvents or non-physiological pH. Such obstacles might be circumvented by using Bri2 BRICHOS based fusion proteins to decorate premade fibers. Amyloid fibrils can self-propagate and potentially also induce other proteins to form amyloid structures^38^. Amyloid-like fibrils and/or oligomers that can be generated from them are potentially cytotoxic^39, 40^ and the seeding and cross-seeding phenomena are thus potentially harmful if amyloid and β-sheet based materials are implanted in living organisms. It can be noted that Aβ_42_ or IAPP amyloid fibrils decorated with recombinant BRICHOS are markedly less prone to seed fibril formation and generate less cytotoxic oligomers compared to the corresponding naïve fibrils^18, 21, 22, 41^. Moreover, addition of recombinant BRICHOS to mouse hippocampal slice preparations in vitro has been shown not to elicit detectable toxic effects and transgenic overexpression of BRICHOS in *Drosophila* fruit flies has no side effects on longevity or locomotor behavior^23, 42, 43^. It is thus conceivable that fibrils that have been decorated with Bri2 BRICHOS based fusion proteins have attenuated capacity to be cytotoxic and therefore can be well tolerated by cells and organisms.

## Experimental procedures

### Plasmids

The synthetic gene coding for Bri2 BRICHOS with mCherry attached to the C-terminal and a hexa-glycine linker between was ordered from GenScript (GenScript Biotech, Netherlands). The pU57 plasmid was digested with *Eco*RI and *Hind*III restriction enzymes and the Bri2 BRICHOS-mCherry gene was isolated on a 2% agarose gel, extracted with QIAquick Gel Extraction Kit (QIAGEN, Venlo Netherlands) and ligated into pT7-H_6_NT*_FlSp_ plasmid that has previously been digested with the same restriction enzymes. To obtain mCherry, HiFi HotStart DNA polymerase (Kapa Biosystems, USA) was used for PCR amplification with the Bri2 BRICHOS mCherry gene as template. The gene was amplified with forward primer 5′-CCG GAA TTC CCT GGT GCC ACG CGG TTC TGT GAG CAA G-3′ and reverse primer 5′-GGG AAG CTT ACT TGT ACA GCT CGT CCA TGC CGC CGG T-3′ at 65 °C as annealing temperature. The PCR product was cleaved and ligated into pT7H_6_NT*_FlSp_ as described above. pET21a-alpha-synuclein (αSN) was a gift from Michael J Fox Foundation MJFF (Addgene plasmid #51486) and was used as template to obtain pT7H_6_NT*_FlSp_(TRS)αSN as described above with the forward primer 5′-GCG GGA ATT CAG AAA ACC TGT ATT TCC AAA TGG ATG TGT TTA TGA AAG G-3′ and reverse primer 5′-CCT GCC AAG CTT ATT ACG CTT CCG GTT CAT AGT CTT G-3′. The plasmid pGEX 2TK encoding for GST-IAPP (kind gift from professor Gunilla Westermark) was used as template to obtain pT7H_6_NT*_FlSp_metIAPP as described above with the forward primer 5′-GCG GAA TTC AAT GAA ATGCAA CAC TGC CAC ATG T-3′ and reverse primer 5′-CCT GCC AAG CTT ACT AAT ATG TAT TGG ATC CCA CG-3′. All the plasmids were transformed into chemically competent *E. coli* Nova Blue cells by heat shock transformation followed by plasmid preparations and sequence verifications. For mammalian expression EGFP of the EGFP-N1 and EGFP-C1 vectors from Clontech were replaced by mCherry. mCherry-C1 was digested with XhoI, isolated on a 1% agarose gel and extracted with QIAquick Gel Extraction Kit (QIAGEN, Venlo Netherlands). Bri2-Brichos was PCR amplified using 5′-AGT CCG GAC TCA GAT CTC GAG CTC AGA CAA TTG AAG AAA ATA TTA AAA TC-3′ as forward and 5′-GCA GAA TTC GAA GCT TGA GCT TAC AGT TTG TAA GTT TCC TTG-3′ as reverse primer, the Phusion DNA polymerase and 58 °C as annealing temperature. The PCR product was purified using the QIAquick PCR purification kit (QIAGEN, Venlo Netherlands). The PCR product and the digested backbone were incubated together with the NEBuilder HiFi DNA Assembly Master Mix (New England Biolabs, Ipswich, USA) and transformed into DH5α *E. coli* bacteria using heat chock transformation (New England Biolabs, Ipswich, USA) followed by plasmid preparations and sequence verifications. An expression plasmid for GFP-tagged N-mutHtt was generated with a PCR fragment generated from pBacMam2-DiEx-LIC-C-FLAG-huntingtin-full-length-Q109 (Addgene #111730) with the Phusion polymerase using forward primer 5′-GTA TTT CCA AAT GGC GAC CCT GGA AAA GCT G-3′ and reverse primer 5′-GTG GAT CCC GTG GTC GGT GCA GCG GCT C-3′, and 71 °C as annealing temperature. The PCR product was used to generate a GFP fusion using NEBuilder HiFi DNA assembly kit (New England Biolabs, Ipswich, USA). For generating Aβ_42_E22G, we used the forward primer 5′-GTT AGA ACC CAC GTC TCC AGC GAA GAA CAC CAG-3′, reverse primer 5′-GTT AGA ACC CAC GTC TCC AGC GAA GAA CAC CAG-3′ and pT7H6NT*_FlSp_ Aβ_42_ as template with the QuikChange II XL site-directed mutagenesis kit (Agilent, US) according to the manufacturers protocol. The pT7H6NT*_FlSp_Aβ_42_E22G plasmid was transformed into chemically competent *E. coli* nova blue cells by heat shock transformation followed by plasmid preparations and sequence verifications.

### Expression and purification protocols

Expression and purification of rh Bri2 BRICHOS-mCherry were done as previously described for rh Bri2 BRICHOS^21^. Aβ42 and β17 were expressed and purified as described previously^25, 30^. Expression of NT*_FlSp_ (TEV recognition site, TRS)-α-synuclein and NT*_FlSp_MetIAPP was performed as described previously^25, 30, 34^. Purification of αSN was performed essentially as described earlier for Aβ^25^. In brief, cell pellets from 1L LB culture were suspended in 8 M urea, 20 mM Tris-HCl pH 8, and sonicated until a clear solution was obtained. The solubilized lysate was passed over a 0.22 μm filter to remove insoluble debris and loaded on a HisPrep FF 16/10 column (Cytiva, Sweden), washed with 15 mM Imidazole, 8 M urea, 20 mM Tris-HCl pH 8 and eluted with 200 mM Imidazole, 8 M urea, 20 mM Tris-HCl pH 8. The eluted NT*_FlSp_(TRS) αSN was dialyzed against 20 mM Tris and cleaved with TEV protease (1:30, TEV:Protein) in 1 mM DTT and 0.2 mM EDTA, 20 mM Tris-HCl pH 8 overnight at 4 °C. The cleaved protein was loaded on a Ni-NTA gravity column to remove NT* and the flow-through was applied on a 3 ml Resource RPC column (Cytiva, Sweden) equilibrated with 0.1% NH_3_ and 2% Acetonitrile. The protein eluted as a single peak using a linear gradient from 2 to 80% Acetonitrile. The peak fractions were pooled, aliquoted to 2 mg per tube, lyophilized and kept at − 20 °C until used. Purification NT*_FlSp_MetIAPP was performed as NT*_FlSp_ (TRS)αSN. NT*_FlSp_MetIAPP were cleaved with 100 mM CNBr at pH 1 by addition om 2 M HCL until final HCl concentration was 0.1 M and incubated at RT overnight. The pelleted IAPP was dissolved in 6 M Guanidine-HCl and applied to 3 ml Resource RPC column (Cytiva, Sweden) equilibrated with 0.1% Trifluoroacetic acid (TFA) and 2% Acetonitrile. The IAPP peptide eluted as a single peak using a linear gradient from 2 to 80% Acetonitrile. The peak fractions were pooled, lyophilized re dissolved in hexafluoroisopropanol (HFIP) at 100 μM and kept at − 20 °C until used.

Aβ_42_E22G was expressed and purified as previously described for Aβ_42_ (Ref.^25^). Synthetic PHF6 (Tau R3 ^306^VQIVYKPVDLSK^317^) was purchased as lyophilized peptide from GenScript (GenScript Biotech, Netherlands) with N- and C-termini capped by acetylation and amidation, respectively.

### Transfection of cells

U2OS and HeLa cells were transiently co-transfected with N-mutHtt109Q-GFP and mCherry-Bri2 BRICHOS or mCherry with Lipofectamine 3000 (Life Technologies), following the manufacture’s instruction. Cells were fixed after 48 h in 4% PFA, stained with Hoechst and mounted with Mowiol.

### Preparation of amyloid fibrils

A volume of 200 μl of 70 μM of Aβ_42_, Aβ_42_E22G, α-synuclein and IAPP was incubated at 37 °C overnight in 20 mM sodium phosphate buffer pH 8 (for Aβ_42_) or 7.4 (for αSN and IAPP) with 10 μM Thioflavin T under quiescent condition in Eppendorf tubes. 100 μM β17 peptide was aggregated by addition of 2.5 mM CaCl_2_ in a volume of 200 μl in Tris-HCl pH 8 with 10 μM Thioflavin T and incubated at 37 °C overnight under quiescent condition. The lyophilized PHF6 peptide was dissolved in 1,1,1,3,3,3-Hexafluoro-2-Propanol (HFIP) to a concentration of 1 mM. 10 μl of HFIP dissolved PHF6 was lyophilized and dissolved in 200 μl, 20 mM ammonium acetate buffer at pH 7 with 200 μM heparin and 10 μM ThT to a final PHF6 concentration of 50 μM and incubated at 37 °C under quiescent condition in Eppendorf tubes overnight. The aggregated peptides were split into two tubes each and centrifuged for 5 min at 21,100× *g*. The supernatant was removed by carefully pipetting over a UV-table to avoid sucking up the pellet. The pellet was resuspended in 100 μl of either 14 μM rh Bri2 BRICHOS-mCherry or 14 μM mCherry (20 μM of each for β17 and 10 μM of each for PHF6), which correspond to 5:1 peptide:BRICHOS ratio and incubated for at least 1 h in 37 °C followed by centrifugation for 5 min at 21,100× *g* and supernatant was removed over UV-table. The pellet was washed twice in 20 mM sodium phosphate buffer, pH 8 (for Aβ_42_ and β17) or 7.4 (for α-synuclein and IAPP) by repeating the procedure above. The final pellet was resuspended in 10 μl sodium phosphate buffer (same as above).

### Fluorescence spectroscopy

Fluorescence emission and excitation scan were measured on a Tecan infinite M100 using Costar 96-wells black flat bottom plate. Both rh Bri2 BRICHOS-mCherry and mCherry were diluted to 5 μM in PBS. In the emission scan, the proteins were excited at 545 nm with 5 nm bandwidth and emission was recorded from 555 to 750 nm with 5 nm bandwidth in steps of 1 nm. In the excitation scan, the protein emission was fixed at 655 nm with 5 nm bandwidth and excitation was scanned over 400–645 nm with 5 nm bandwidth in steps of 1 nm.

### Thioflavin T (ThT) fluorescence measurements of Aβ42 fibril formation

Aβ_42_ aggregation under quiescent conditions were recorded by ThT fluorescence as described previously^23^ using a POLARstar Omega plate reader (BMG Labtech, Germany). Measurements were carried out of 20 µl per well, containing Aβ_42_ monomer only, or Aβ_42_ monomer plus isolated monomers of rh Bri2 BRICHOS-mCherry or rh Bri2 BRICHOS, 10 μM ThT in 0.02% NaN3, 0.2 mM EDTA and 20 mM sodium phosphate, pH 8.0. Traces shown are averages of 4 replicates with standard deviations as error bars.

### Fluorescence microscopy

Transfected cells were imaged using a Zeiss LSM 880 microscope equipped with a plan-Apo 63X/1.40 Oil objective. Line scan analysis was performed using ImageJ^44^. The values of each line scan was normalized to its maximum value. For quantifying the percentage of cells that displayed co-localization of mCherry-Bri2 BRICHOS or mCherry at N-mutHttQ109-GFP inclusions, cells were scored manually from three independent experiments (n = 35–50). Four μl of resuspended pelleted amyloid fibrils of Aβ_42,_ α-synuclein, IAPP, Aβ_42_E22G, PHF6, or β17 were transferred to a microscope slide (Thermo Scientific, USA) containing Vectashield mounting medium for fluorescence and cover slip was carefully mounted. Fluorescence images were collected with an EVOS FL Auto 2 imaging system (Invitrogen, USA) using GFP-channel (ex:470/22 nm; em:510/42 nm) to visualize Thioflavin T fluorescence and TX Red-Channel (ex:585/29 nm; em:624/40 nm) to visualize mCherry fluorescence. Fluorescence intensities were measured with ImageJ software^44^.

### Immuno EM

The amyloid fibril was incubated with mCherry or rh Bri2 BRICHOS-mCherry for 60 min at 37 °C in the same conditions as above. It was then centrifuged at 12,000 rpm for 10 min to pellet down the fibrils. The supernatant was discarded, and the pellet was washed 3 times with sodium phosphate buffer, and it was diluted to 10 µM for EM sample preparation. 5 µl diluted solution was applied on 200 mesh formvar coated nickel grid and excess solution was removed using blotting paper after 10 min of incubation. It was then washed twice with 10 µl MQ water. The nickel grid surface was blocked with 1% bovine serum albumin (prepared in PBS) for 30 min. It was then washed thrice with MQ water. 5 µl primary anti-RFP antibody (RF5R monoclonal antibody, Thermo Fisher Scientific, USA, 1:200 dilution in PBST) was applied to the grid and it was incubated for 60 min at room temperature. Then, the grids were washed thrice with MQ water. Further, the nickel grids were incubated with anti-mouse IgG-gold (BBI Solutions, Crumlin, UK) (1:40 dilution in PBST) secondary antibody for 60 min. Extra solutions were removed and then it was washed thrice with MQ water and then stained with 1% uranyl formate for 5 min. Extra stained was blotted with blotting paper and it was air-dried. Transmission electron microscopy (FEI Tecnai 12 Spirit BioTWIN, operated at 100 kV) was performed for analysis of fibril morphology using 2 k × 2 k Veleta CCD camera (Olympus Soft Imaging Solutions, GmbH, Münster, Germany) 0.15–20 images were recorded for each sample randomly. Images were obtained at a magnification of × 43,000 and × 87,000. For the manuscript × 87,000 magnified images were used.

### Surface plasmon resonance

Binding of Bri2 BRICHOS-mCherry and mCherry to Aβ_42_ fibrils was measured with a BIACORE 3000 instrument. Sonicated Aβ_42_ fibrils were immobilized on a CM5 sensor chips (Cytiva) using standard amine-coupling chemistry. A fresh mixture of 0.05 M NHS and 0.2 M EDC was added to the sensor-chip surface for activation, followed by incubation with 5.6 μM Aβ_42_ diluted in 10 mM sodium acetate pH 4.5 and finally deactivation by ethanolamine. Blank channels for negative controls were prepared by omitting protein in the coupling step. Each experiment involved five different protein concentrations of either 313 nM–40 μM mCherry or 39 nM–5 μM Bri2 BRICHOS-mCherry and subsequent buffer flow to monitor dissociation. The experiments were performed with HBS-E (10 mM HEPES, 150 mM NaCl, 0.2 mM EDTA, pH 7.4) as running buffer and a flow rate of 25 μL/min. The chip surface was regenerated between each sample by injection of 30 mM NaOH for 30 s. Analysis of the SPR data was done as previously described^33^.

## Supplementary Information

Supplementary Figures.

## Data Availability

Data are available from the corresponding author upon request.

## References

[1] Dobson CM (2003). Protein folding and misfolding. Nature.

[2] Knowles TP, Buehler MJ (2011). Nanomechanics of functional and pathological amyloid materials. Nat. Nanotechnol..

[3] Meyers MA, McKittrick J, Chen PY (2013). Structural biological materials: critical mechanics-materials connections. Science.

[4] Benson MD (2018). Amyloid nomenclature 2018: recommendations by the International Society of Amyloidosis (ISA) nomenclature committee. Amyloid.

[5] Christensen LFB, Schafer N, Wolf-Perez A, Madsen DJ, Otzen DE (2019). Bacterial amyloids: biogenesis and biomaterials. Adv. Exp. Med. Biol..

[6] Rising A, Johansson J (2015). Toward spinning artificial spider silk. Nat. Chem. Biol..

[7] Eisenberg DS, Sawaya MR (2017). Structural studies of amyloid proteins at the molecular level. Annu. Rev. Biochem..

[8] Iadanza MG, Jackson MP, Hewitt EW, Ranson NA, Radford SE (2018). A new era for understanding amyloid structures and disease. Nat. Rev. Mol. Cell Biol..

[9] Kollmer M (2019). Cryo-EM structure and polymorphism of Abeta amyloid fibrils purified from Alzheimer's brain tissue. Nat. Commun..

[10] Landreh L, Sawaya MR, Hipp MS, Eisenberg DS, Wüthrich K, Hartl FU (2016). The formation, function and regulation of amyloids: insights from structural biology. J. Intern. Med..

[11] Knowles TP, Mezzenga R (2016). amyloid fibrils as building blocks for natural and artificial functional materials. Adv. Mater..

[12] Eisenberg D, Jucker M (2012). The amyloid state of proteins in human diseases. Cell.

[13] Buxbaum J, Johansson J (2017). Transthyretin and BRICHOS: the paradox of amyloidogenic proteins with anti-amyloidogenic activity for Aβ in the central nervous system. Front. Neurosci..

[14] Kallberg Y, Gustafsson M, Persson B, Thyberg J, Johansson J (2001). Prediction of amyloid fibril-forming proteins. J. Biol. Chem..

[15] Szyperski T (1998). Pulmonary surfactant-associated polypeptide C in a mixed organic solvent transforms from a monomeric alpha-helical state into insoluble beta-sheet aggregates. Protein Sci..

[16] Willander H (2012). High-resolution structure of a BRICHOS domain and its implications for anti-amyloid chaperone activity on lung surfactant protein C. Proc. Natl. Acad. Sci. USA.

[17] Nerelius C, Gustafsson M, Nordling K, Larsson A, Johansson J (2009). Anti-amyloid activity of the C-terminal domain of proSP-C against amyloid beta-peptide and medin. Biochemistry.

[18] Oskarsson ME (2018). The BRICHOS domain of Bri2 inhibits islet amyloid polypeptide (IAPP) fibril formation and toxicity in human beta-cells. Proc. Natl. Acad. Sci. USA.

[19] Willander H (2012). BRICHOS domains efficiently delay fibrillation of amyloid beta-peptide. J. Biol. Chem..

[20] Arosio P (2016). Kinetic analysis reveals the diversity of microscopic mechanisms through which molecular chaperones suppress amyloid formation. Nat. Commun..

[21] Chen G (2017). Bri2 BRICHOS client specificity and chaperone activity are governed by assembly state. Nat. Commun..

[22] Cohen SI (2015). A molecular chaperone breaks the catalytic cycle that generates toxic *Abeta oligomers*. Nat. Struct. Mol. Biol..

[23] Poska H (2016). Dementia related Bri2 BRICHOS is a versatile molecular chaperone that efficiently inhibits Abeta42 toxicity in Drosophila. Biochem. J..

[24] Shaner NC (2004). Improved monomeric red, orange and yellow fluorescent proteins derived from *Discosoma* sp. red fluorescent protein. Nat. Biotechnol..

[25] Abelein A (2020). High-yield production of amyloid-beta peptide enabled by a customized spider silk domain. Sci. Rep..

[26] MacDonald MEEA (1993). A novel gene containing a trinucleotide repeat that is expanded and unstable on Huntington's disease chromosomes. The Huntington's Disease Collaborative Research Group. Cell.

[27] Davies SW (1997). Formation of neuronal intranuclear inclusions underlies the neurological dysfunction in mice transgenic for the HD mutation. Cell.

[28] Bauerlein FJB (2017). In situ architecture and cellular interactions of PolyQ inclusions. Cell.

[29] Sanchez-Pulido L, Devos D, Valencia A (2002). BRICHOS: a conserved domain in proteins associated with dementia, respiratory distress and cancer. Trends Biochem. Sci..

[30] Sarr M (2018). A spidroin-derived solubility tag enables controlled aggregation of a designed amyloid protein. FEBS J..

[31] Wood SJ (1999). Alpha-synuclein fibrillogenesis is nucleation-dependent. Implications for the pathogenesis of Parkinson's disease. J. Biol. Chem..

[32] Nilsberth C (2001). The 'Arctic' APP mutation (E693G) causes Alzheimer's disease by enhanced Abeta protofibril formation. Nat. Neurosci..

[33] Rahman MM, Zetterberg H, Lendel C, Hard T (2015). Binding of human proteins to amyloid-beta protofibrils. ACS Chem. Biol..

[34] Kronqvist N (2017). Efficient protein production inspired by how spiders make silk. Nat. Commun..

[35] Rising A, Widhe M, Johansson J, Hedhammar M (2010). Spider silk proteins: recent advances in recombinant production, structure-function relationships and biomedical applications. Cell. Mol. Life Sci..

[36] Salehi S, Koeck K, Scheibel T (2020). Spider silk for tissue engineering applications. Molecules.

[37] Jansson R (2014). Recombinant spider silk genetically functionalized with affinity domains. Biomacromol.

[38] Tjernberg L, Rising A, Johansson J, Jaudzems K, Westermark P (2016). Transmissible amyloid. J. Intern. Med..

[39] Bucciantini M (2004). Prefibrillar amyloid protein aggregates share common features of cytotoxicity. J. Biol. Chem..

[40] Bucciantini M (2002). Inherent toxicity of aggregates implies a common mechanism for protein misfolding diseases. Nature.

[41] Chen G (2020). Augmentation of Bri2 molecular chaperone activity against amyloid-beta reduces neurotoxicity in mouse hippocampus in vitro. Commun. Biol..

[42] Hermansson E (2014). The chaperone domain BRICHOS prevents CNS toxicity of amyloid-beta peptide in Drosophila melanogaster. Disease Models Mech..

[43] Kurudenkandy FR (2014). Amyloid-beta-induced action potential desynchronization and degradation of hippocampal gamma oscillations is prevented by interference with peptide conformation change and aggregation. J. Neurosci..

[44] Schneider CA, Rasband WS, Eliceiri KW (2012). NIH image to ImageJ: 25 years of image analysis. Nat. Methods.

