# Supplementary material for: Functionalization of amyloid fibrils via the Bri2 BRICHOS domain
Source: Sci Rep. 2020 Dec 10;10:21765. doi: 10.1038/s41598-020-78732-1 (PMC7730125; doi:10.1038/s41598-020-78732-1)
Supplement: Supplementary file 1 — Supplementary Figures. [file 41598_2020_78732_MOESM1_ESM.docx]

Supplementary Information

**Functionalization of amyloid fibrils via the Bri2 BRICHOS domain**

**Henrik Biverstål^1,2^, Rakesh Kumar^1^, Anna Katharina Schellhaus^3^, Médoune Sarr^1^, Nico P. Dantuma^3^, Axel Abelein^1^ and Jan Johansson^1^***

^1^Department of Biosciences and Nutrition, Karolinska Institutet, Neo, 141 83 Huddinge, Sweden.

^2^Department of Physical Organic Chemistry, Latvian Institute of Organic Synthesis, Riga LV-1006, Latvia

^3^Department of Cell and Molecular Biology, Karolinska Institutet, 171 77 Stockholm, Sweden.

**
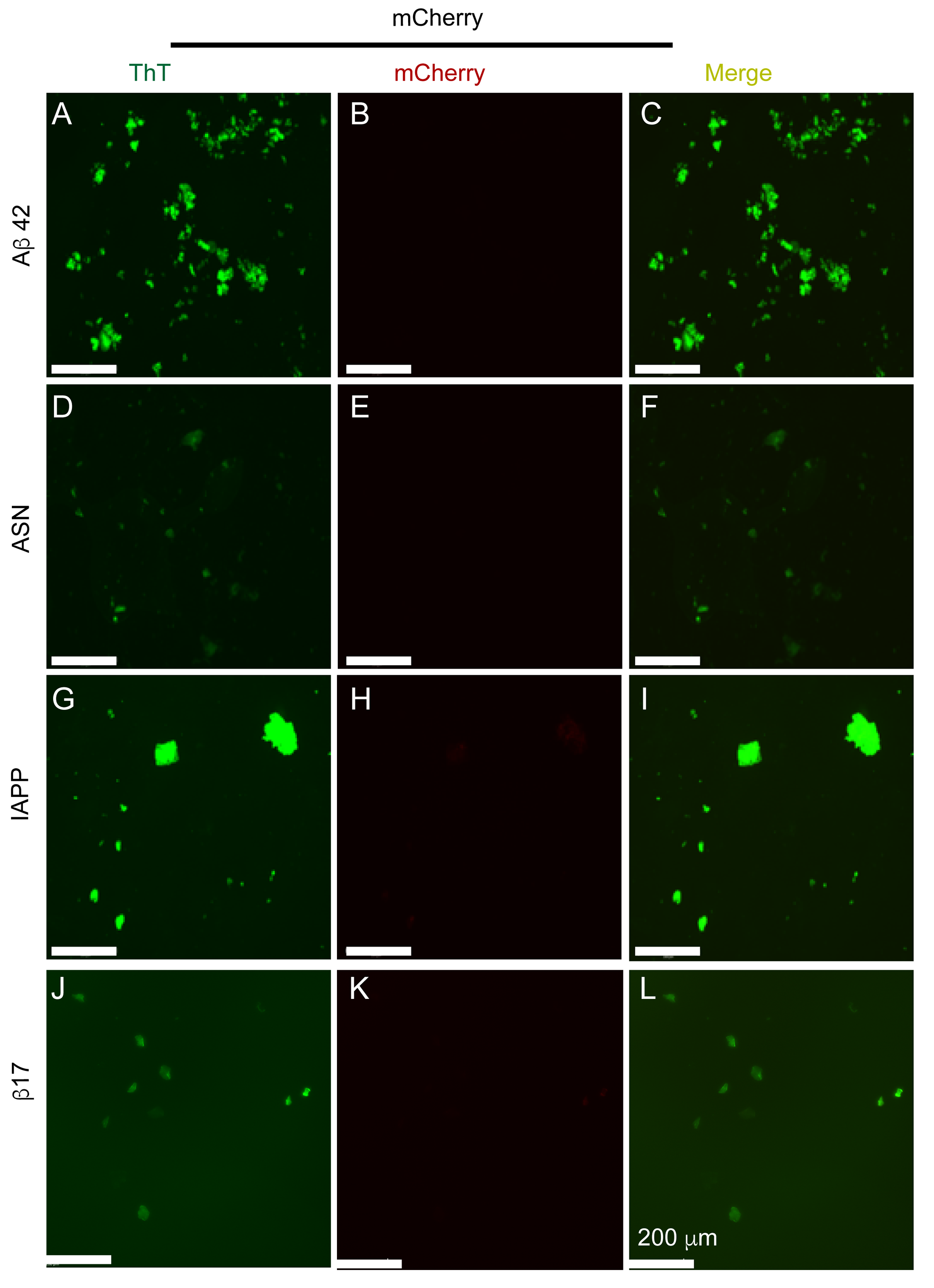
**

**Supplementary** **Figure 1.** mCherry binding to amyloid fibrils. (A,D,G,J) ThT staining of fibrillated Aβ_42_ (A), α-synuclein (ASN) (D), IAPP (G) and β17 (J). (B,E,H,K) fluorescence of mCherry incubated with fibrillated Aβ_42_ (B), ASN (E), IAPP (H) and β17 (K). (C,F,I,L) merged images of ThT and mCherry signals.

**Supplementary** **Figure 2.** Bri2 BRICHOS-mCherry and mCherry binding to Aβ_42_E22G and PHF6. (A,D,G,J,) ThT staining of fibrillated Aβ_42_E22G (A,G), PHF6 (D,J). (B,E,H,K) fluorescence of Bri2 BRICHOS-mCherry incubated with fibrillated Aβ_42_E22G (B), PHF6 (E) and fluorescence of mCherry incubated with fibrillated Aβ_42_E22G (H), PHF6 (K). (C,F,I,L) merged images of ThT and mCherry signals.

**Supplementary Figure 3.** SPR sensorgrams used to determine *K*_D_ values for Bri2 BRICHOS-mCherry (A) and mCherry (B) binding to immobilized Aβ_42_-fibrils. Bri2 BRICHOS-mCherry was injected in the concentration range 39-625 nM and mCherry was injected in the concentration range 2.5-40 μM.
